# Microstructure and Mechanical Properties of Core-Shell B_4_C-Reinforced Ti Matrix Composites

**DOI:** 10.3390/ma16031166

**Published:** 2023-01-30

**Authors:** Ziyang Xiu, Boyu Ju, Junhai Zhan, Ningbo Zhang, Pengjun Wang, Keguang Zhao, Mingda Liu, Aiping Yin, Weidi Chen, Yang Jiao, Hao Wang, Shuyang Li, Xiaolin Zhu, Ping Wu, Wenshu Yang

**Affiliations:** 1 State Key Laboratory of Advanced Welding and Jointing, Harbin Institute of Technology, Harbin 150001, China; 2 Shanghai Aerospace System Engineering Research Institute, Shanghai 201108, China; 3 Aerospace Research Institute of Materials & Processing Technology, Beijing 100076, China; 4 Xi’an Honor Device Co., Ltd., Xi’an 710000, China; 5 Huazhong Institute of Electro-Optics, Wuhan 430074, China; 6 CASIC Space Engineering Development Co., Ltd., Xinzhou 431400, China; 7 Key Laboratory of Advanced Science and Technology on High Power Microwave, Xi’an 710024, China; 8 Northwest Institute of Nuclear Technology, Xi’an 710024, China

**Keywords:** B_4_C, Ti matrix composite, core-shell composite, mechanical properties

## Abstract

Composite material uses ceramic reinforcement to add to the metal matrix to obtain higher material properties. Structural design is an important direction of composite research. The reinforcement distribution of the core-shell structure has the unique advantages of strong continuity and uniform stress distribution. In this paper, a method of preparing boron carbide (B_4_C)-coated titanium (Ti) powder particles by ball milling and preparing core-shell B_4_C-reinforced Ti matrix composites by Spark Plasma Sintering was proposed. It can be seen that B_4_C coated on the surface of the spherical Ti powder to form a shell structure, and B_4_C had a certain continuity. Through X-ray diffraction characterization, it was found that B_4_C reacted with Ti to form layered phases of titanium boride (TiB) and titanium carbide (TiC). The compressive strength of the composite reached 1529.1 MPa, while maintaining a compressive strain rate of 5%. At the same time, conductivity and thermal conductivity were also characterized. The preparation process of the core-shell structure composites proposed in this paper has high feasibility and universality, and it is expected to be applied to other ceramic reinforcements. This result provides a reference for the design, preparation and performance research of core-shell composite materials.

## 1. Introduction

Compounding is an important material design method, which uses ceramic (such as SiC, B_4_C, AlN, Si_3_N_4_, etc.) reinforcement to compound with a metal matrix to obtain higher material properties [1,2,3]. B_4_C is an ideal reinforcement with a light weight, high hardness and high elastic modulus [4,5]. It is used in metal matrix composites to greatly improve the mechanical properties. Based on the excellent properties of B_4_C, high-strength and high-wear resistant composites have been developed and applied in the military, automotive and nuclear industries [6,7]. 

The reinforcement of traditional B_4_C/metal composites is mainly B_4_C particles (B_4_C_p_). Researchers have carried out structural design research on B_4_C_p_/Al and B_4_C_p_/Ti matrix composites by adjusting the volume fraction, morphology and dispersion of B_4_C. Luo and Zhang et al. [8,9] found that increasing the B_4_C_p_ content could effectively improve the properties, but when the mass fraction was 27.5%, B_4_C_p_ agglomerated significantly, resulting in no further improvement in strength. Wu et al. [10] found that when the volume fraction was fixed, the smaller the B_4_C_p_ diameter, the higher the strength. Small-size B_4_C_p_ can effectively lead to larger values in strain gradient strengthening as well as CTE mismatch strengthening. Zhang et al. [11] prepared B_4_C_p_/TiAl composites with different mass fractions. It was found that the flexural strength and fracture toughness of 20 wt.% B_4_C_p_/TiAl were significantly improved compared with 10 wt.%. Selvakumar et al. [12] prepared 10 wt.% B_4_C_p_/Ti6Al4V composites and found that the hardness of the composites increased with the increase of the ball milling time. Chen et al. [13] prepared 30 wt.% B_4_C_p_/6061Al composites by hot pressing-extrusion-rolling. The high-volume fraction B_4_C_p_ was uniformly dispersed, and the tensile strength of the composite reached 265 MPa. B_4_C/Ti composites were prepared by a laser engineered net-shaping process by Nartu et al. [14], and the microscopic process of TiB and TiC formed by the reaction of B_4_C and Ti was studied and analyzed. 

A new study found that the powder with a core-shell structure has unique performance characteristics when sintered. The shell structure is connected into a network after sintering, which can effectively transfer the load and exert excellent mechanical properties. Yang et al. [15,16,17] oxidized Ti6Al4V titanium alloy powder at a high temperature to prepare a core-shell structure with the oxide of Ti6Al4V as the core and titanium as the shell. The titanium alloy powder with the core-shell structure was sintered into titanium matrix composites by spark plasma sintering. It was found that this structure has good oxidation resistance and high temperature stability. Li et al. [18] adsorbed B_4_C on the surface of spherical Ti powder to prepare Ti-B_4_C particles with a core-shell structure, and then reinforced 2024Al alloy. It was found that the yield strength of 10 wt.% Ti-B_4_C/2024Al composites increased by 37.2% and the elongation increased by 6.3%. The excellent performance was attributed to the combined effect of the Ti particles, B_4_C particles and in situ TiAl_3_ phase. Zygula et al. [19] used B_4_C to react with β-Ti alloy in situ to form TiB and TiC, and clarified the diffusion and reaction behavior of alloying elements. Jiang et al. [20] studied the reaction process of B_4_C and Ti during SPS reaction. The products were mainly TiC, and a small amount of TiB and TiB_2_. However, the current research on B_4_C/Ti composites is mostly focused on particle-reinforced metal matrix composites. The distribution of B_4_C is dispersed, and there is no preparation method for core-shell B_4_C/Ti composites. Furthermore, the effect of the shell-like distribution of B_4_C on the properties of composites is not clear.

In this paper, Ti powder was used as the core and granular B_4_C as the shell. The research on the preparation of composite materials with core-shell Ti-B_4_C powder was carried out. The dispersion and preparation processes were optimized to guide the preparation of core-shell structural materials. The particularity of the core-shell structure and its unique mechanical properties were studied.

## 2. Materials and Methods

### 2.1. Raw Materials

**Figure 1:**
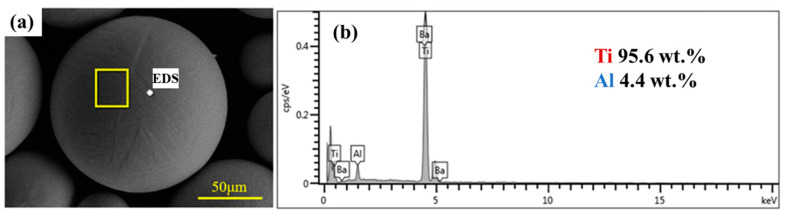
Raw material characterization of Ti powder. (**a**) Morphological characterization by SEM. (**b**) Element content characterization by EDS.

**Figure 2:**
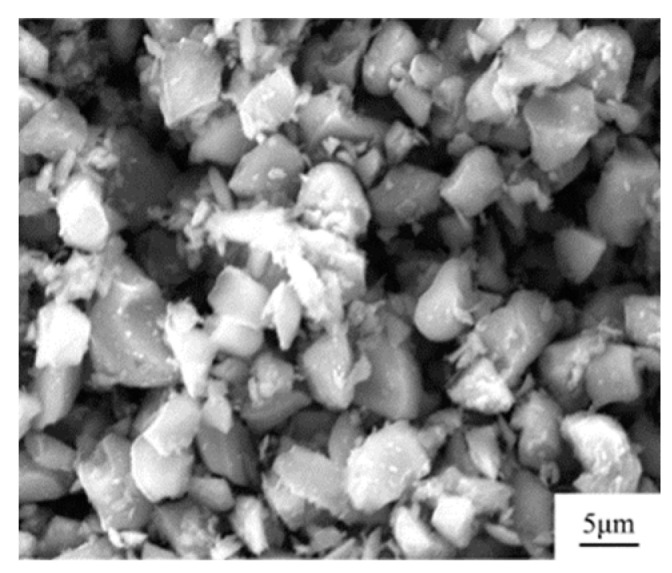
Raw material characterization of B_4_C powder.

The Ti powder used in this project was high-purity titanium powder, which was supplied by the Northwest Institute of Nonferrous Metals, China. In addition, its morphology was a spherical titanium powder with a larger particle size through plasma spheroidization. The energy spectrum analysis of the original spherical Ti powder was carried out. The experimental results are shown in Figure 1. It can be seen that the purity of Ti powder was high. Ti powder particle size distribution was in the range of 40~100 μm, and the shape in a better spherical, enlarged observation of its surface can be found on the surface of a smooth, not foreign matter, which also led to the powder having good fluidity. B_4_C powders were supplied by Nangong Jingrui Alloy, China. The morphology of the original B_4_C particles is shown in Figure 2. The average diameter of Ti powder used was 80 μm and that of B_4_C powder was 10 μm. 

**Figure 3:**
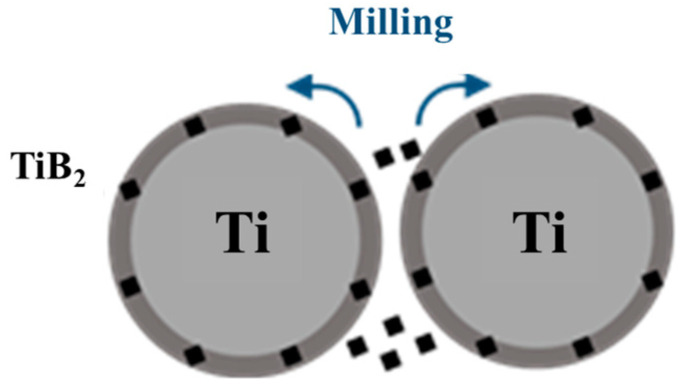
Preparation of core-shell Ti-B_4_C composite by ball milling.

In this paper, the precursor particles (B_4_C) were uniformly coated on the surface of the Ti powder by mechanical ball milling to form a core-shell structure of the B_4_C precursor shell-coated Ti powder, as shown in Figure 3. B_4_C coating on the surface of the Ti powder was achieved by mechanical ball milling. The volume fraction of B_4_C was 30% in ball milling. The equipment used was the planetary ball mill apparatus QM-3SP2, from the Instrument Factory of Nanjing University, China. The mill and the ball used in the ball mill were both made of alumina. The diameter of the ball was 3 mm, and the volume of the ball mill was 500 mL. The rotation speed was 250 r/min, the ball milling time was 8 h and the ball milling atmosphere was ball milling under argon protection. The ball to material ratio was 5:1. 

### 2.2. Preparation of Ti-Based Composites with a Core-Shell Microstructure

The core-shell structured powders were prepared by the Spark Plasma Sintering (SPS) process. SPS is a new material-sintering technology, which is widely used in the research and development of composite materials because of its fast-heating rate, short sintering time, controllable structure, energy saving and environmental protection [21,22]. After the powder coating process, 110 g mixed powder was stacked into high-density graphite die with an internal diameter of 50 mm. Then, sintering was performed on the SPS furnace (FCT HPD-250, Germany, Rauenstein) under a vacuum environment. For the sintering temperature, please reference the Ti alloy preparation temperature [23,24]. In this paper, the core-shell structure powder was continuously sintered at 1200 °C for 35 min. The sintering pressure, soaking time and vacuum were maintained at 40 MPa, 15 min and <8 Pa, respectively. After sintering, sintered composites were furnace-cooled to room temperature and the pressure was removed at 600 °C.

### 2.3. Microstructure Characterization of Ti-Based Composites

The phase composition of both the mixed powders and the composites were characterized by an Empyrean Intelligent X-ray Diffractometer (Malvern Panalytical, Malvern, UK). The specific test conditions were as follows: accelerating voltage 40 kV, current 40 mA, Cu-Kα radiation, scanning speed 10°/min and scanning angle range 10~90°. Before the collection of the diffraction patterns, the tested powder was evenly and randomly laid on the glass test platform, and the surface of the tested composite block was sandpapered and cleaned with an acetone solution.

The microstructure of the mixed powder and the composites were observed and snapped by a ZEISS459315 (Carl Zeiss A.G., Oberkochen, Cermany) metallographic microscope and Quanta 200FEG (FEI Company, Hillsboro, OR, USA) field-emission scanning electron microscopy (SEM) equipped with energy dispersive spectrometer (EDS). The sample with a dimension of 4 mm × 5 mm × 3 mm was obtained by electro discharge wire cutting. Before this, the observed samples were successively polished, cleaned and etched (400 #, 800 #, 2000 # and 4000 # sandpaper were selected for polishing, and a diamond polishing agent was selected for polishing cloth; the etched solution was Kroll reagent with a ratio of 20 vol%HF + 20 vol%HNO_3_ + 60 vol%H_2_O).

### 2.4. Performance Tests of Ti-Based Composites

#### 2.4.1. Compression Test

The compression test was carried out on an Instron-8862 (Instron, Norwood, MA, USA) universal electronic testing machine with a constant displacement velocity of 0.25 mm/min for the indenter of the machine. To avoid defects from adversely affecting the compression test results, the test samples with a dimension of 4 mm × 4 mm × 6 mm were ground with 1500 # sandpaper until the surface had no obvious macroscopic defects. The compressive strength (*P*) of the composite could be estimated by:

(1) $$P=\frac{F}{S}$$

where *F* is the maximum load when the specimen is fractured in compression and *S* denotes the cross-sectional area of the specimen perpendicular to the direction of the load. All samples were tested repeatedly more than five times to ensure the stability of the results.

#### 2.4.2. Thermal Conductivity Measurement

The cylindrical specimen with the size of *Φ*12.7 mm × 3.2 mm was processed by the wire-cutting method, and then the upper and lower surfaces of the specimen were polished with 1000 # sandpaper to ensure a smooth and flat surface. To ensure that the surface of the specimen was evenly heated during the thermal conductivity measurement, the upper and lower surfaces of the specimen were evenly coated with carbon powder after polishing. The thermal conductivity test experiments were performed on an LFA-447 laser thermal conductivity meter manufactured by NETZSCH, which tests the thermal diffusion coefficient *k* of composite specimens at room temperature. The thermal conductivity *λ* of the composites could be obtained from:

(2) λ=k×ρ×C

where *k*, *ρ* and *C* are the thermal diffusion coefficient, density and thermal capacity of the composites. *ρ* was obtained by Archimedes method and *C* was evaluated by the law of mixing.

## 3. Results and Discussion

### 3.1. Microstructure Characterization of Core-Shell Ti-B_4_C Particles

**Figure 4:**
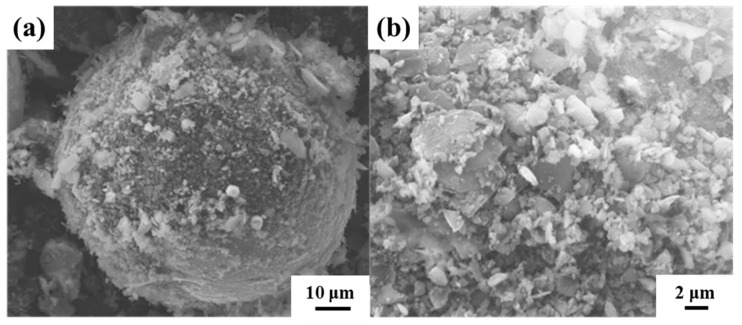
SEM characterization of core-shell Ti-B_4_C (30 vol.%). (**a**) Core-shell Ti-B_4_C, (**b**) the local amplification results of (**a**).

Using the ball milling process parameters determined above, the large-sized spherical titanium powder and small-sized B_4_C particles were ball milled to prepare a core-shell structure with spherical titanium powder as the core and B_4_C as the shell. In order to prepare a thicker shell, the B_4_C volume fraction was selected to be 30%. The surface morphology of the core-shell structure formed after ball milling is shown in Figure 4. It can be seen that the surface of the spherical titanium powder was obviously coated, but the uniformity was poor. The spherical titanium powder had a slight deformation in shape, but it was still spherical on the whole. After further magnification observation, compared with the original spherical titanium powder, it can be found that the spherical titanium powder particles were no longer smooth on the surface. Due to the high-volume fraction of the reinforcement precursor, some precursors were agglomerated on the surface, and many small particles were distributed on the surface.

**Figure 5:**
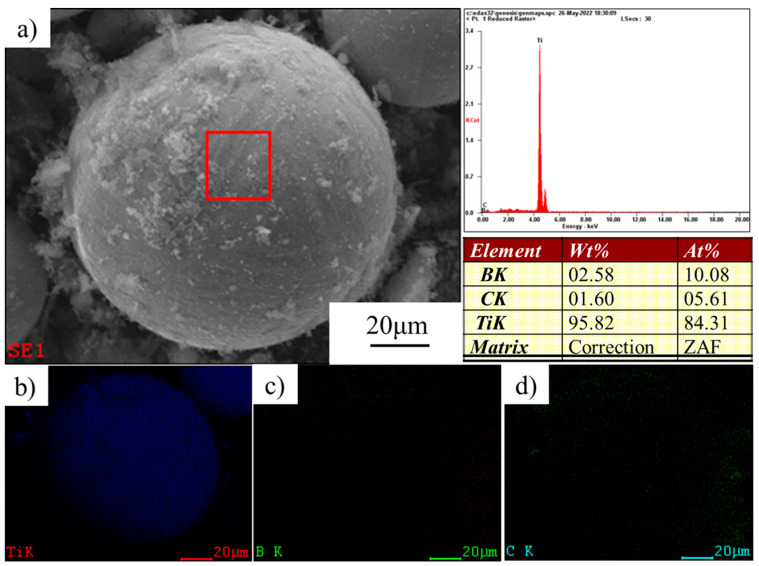
(**a**) EDS characterization of core-shell Ti-B_4_C structure; (**b**–**d**) surface scanning analysis of Ti, B and C elements, respectively.

In order to analyze the composition distribution, the energy spectrum characterization of the core-shell structure Ti-B_4_C was carried out, and the results are shown in Figure 5. It can be seen that the main component of the spherical particles was the Ti element, and a small amount of B and C elements were distributed on the surface, corresponding to the B_4_C particles added by ball milling. It can be seen that B_4_C particles were dispersed on the surface of the Ti powder after ball milling, forming the microstructure of B_4_C-coated Ti. A discontinuous B_4_C shell was formed on the surface of the Ti powder after sintering.

### 3.2. Microstructure Characterization of Ti-B_4_C Composites

**Figure 6:**
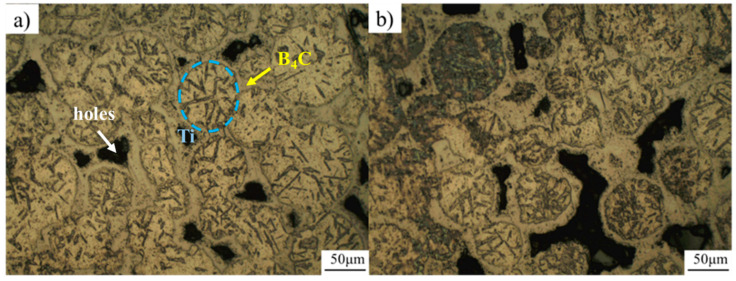
Metallographic characterization of Ti-B_4_C composites; (**a**,**b**) are the representation of different regions.

**Figure 7:**
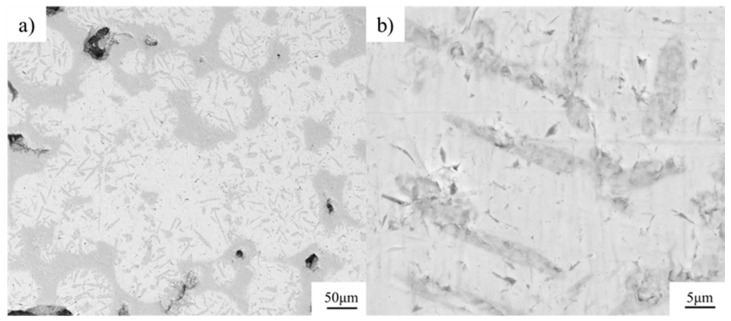
SEM backscattering of Ti-B_4_C composites; (**b**) is the local amplification of (**a**).

The Ti-B_4_C core-shell structure was sintered by SPS with the parameters of 1200 °C −35 min and a preset pressure of 40 MPa. The metallographic structure is shown in Figure 6. The sintered composite formed a clear network structure, but there were obvious holes between the powder particles, and the density of the material was low. The composites were observed by SEM, as shown in Figure 7. It can be seen that the core-shell structure unit based on the spherical Ti powder was retained, and the precursor particles coated with the spherical Ti powder formed a network reinforcement during the sintering process and bonded well with the matrix interface. 

The XRD phase compositions of the Ti-B_4_C core-shell structure before sintering, after ball milling and the as-sintered composite were compared and analyzed. The results are shown in Figure 5. It can be seen that the reaction occurred during the ball milling process to generate TiB and TiC. After sintering, the phases were still dominated by Ti, B_4_C, TiC and TiB, but the intensity of the characteristic peak of TiC increased, indicating that the interfacial reaction between B_4_C and Ti further increased during sintering. A similar reaction process was found in the conventional particulate B_4_C/Ti composites [14,25]. 

**Figure 8:**
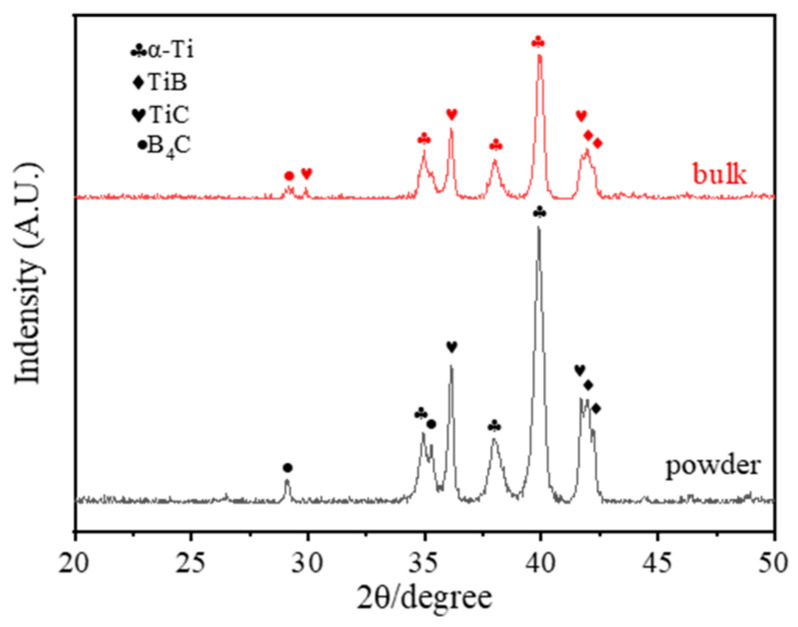
XRD characterization of Ti-B_4_C core-shell structure powder and composite.

From the back-scattering characterization results of Figure 7, it can be seen that the Ti element was spherically distributed, while the light elements (B and C) were distributed on the surface of Ti, forming a shell structure. This structure was consistent with previous research results. The interfacial reaction between B_4_C and Ti occurred, and a small amount of B_4_C decomposed to form TiB and TiC, as shown in XRD (Figure 8). The needle-like TiB and TiC phase was distributed from the surface of Ti particles to the inside of the Ti particles. The B and C atoms provided by B_4_C were diffused from the core-shell structure shell to the core, thus forming a staggered lamellar reinforcement inside the core-shell structure unit, and the results are shown in Figure 7.

### 3.3. Mechanical and Functional Properties of Ti-B_4_C Composites

The ability of a material to withstand axial static pressure at room temperature reflects the ability of the material to resist deformation during application, which depends on the type of reinforcement, interfacial bond strength, reinforcement distribution pattern and reinforcement content of the core-shell composite.

**Figure 9:**
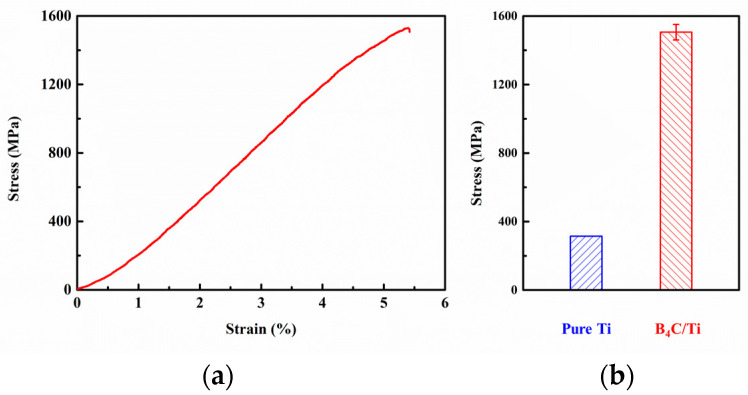
Compressive mechanical properties of composites with core-shell structure; (**a**) strain–stress curves; (**b**) comparison of mechanical properties (the yield strength of pure Ti is obtained from ref. [26]).

Figure 9 demonstrates the compressive stress–strain curves of the three core-shell structure composites B_4_C/Ti. It can be seen that the plastic deformation phase was not obvious in the stress–strain curves of the three composites, which proves that the introduction of a large number of brittle reinforcements significantly increases the brittleness of the composites. For the B_4_C/Ti composites, the interfacial reaction between B_4_C and Ti produced TiB and TiC, forming a better interfacial bond. The good interfacial bonding strength resulted in the B_4_C/Ti core-shell structure composite with a compressive strength of 1529.1 MPa. Compared to the compressive strength of the Ti matrix, the yield strength of all three composites was significantly improved. Typically, the B_4_C/Ti composite has a 3.8-times improvement in the yield strength, as shown in Table 1.

**Figure 10:**
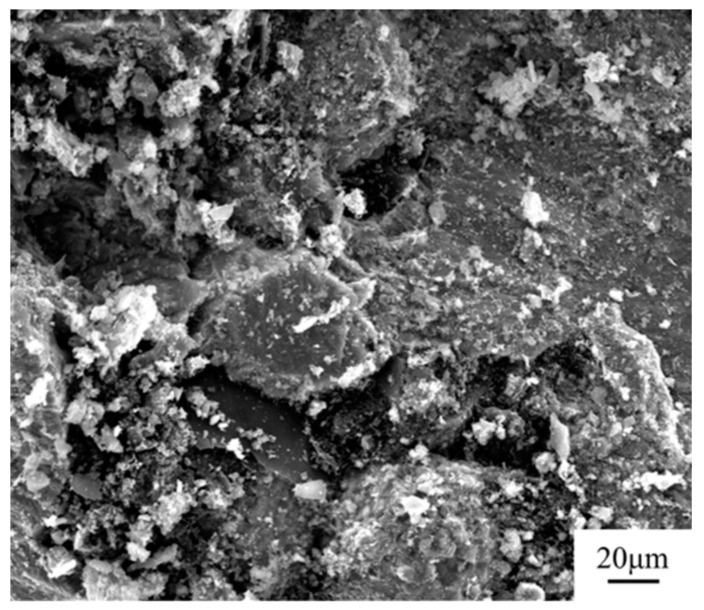
Fractured surface morphology of B_4_C/Ti composites with core-shell structure.

Figure 10 shows the room-temperature compression fracture morphology of B_4_C/Ti core-shell structure composites. It can be seen that the fractures’ surfaces of B_4_C/Ti composites were uneven, which suggest that the crack expansion path in the composite was increased, causing the composite to absorb more energy before the fracture. Therefore, the compressive strain rate of more than 5% is still maintained at a high B_4_C volume fraction (30 vol.%).

In addition, the hardness, thermal conductivity and electric conductivity of the B_4_C/Ti composites were tested, and the results are shown in Table 2. The hardness of the B4C/Ti composites reached a high level (697.89 HV), which indicates that the B_4_C reinforcement has a more favorable strengthening effect. At the same time, due to the influence of pores, the electrical conductivity and thermal conductivity of the composites were low. The test results of this material provide a reference for subsequent core-shell material designs.

## 4. Conclusions

In this paper, Ti-B_4_C core-shell structure composites were prepared by ball milling and SPS. The mechanical properties, electrical conductivity and thermal conductivity of the composites were studied. B_4_C reacts with Ti to form TiB and TiC, and obvious pores and defects are observed in the composites. The compressive strength of core-shell B_4_C/Ti composites is up to 1529.1 MPa, which, compared with the Ti matrix, has a substantial increase, while the material maintains a compressive strain of 5%. This result has reference significance for the preparation of core-shell B_4_C/Ti composites.

## Figures and Tables

**Table 1:** The mechanical properties of B_4_C/Ti composites were reported.

	Composite	Mechanical Properties
Zhang [11]	30 wt.% B_4_C/TiAl	Bending 437.3 MPa
Yang [15,16]	(TiB, TiC, Nd_2_O_3_)/Ti	Bending 1150 MPa
Choi [25]	20 wt.% B_4_C/Ti	Tensile 699 MPa
Wu [27]	Monolithic B_4_C/Ti	Flexural 496.2 MPa
Han [28]	1 wt.% B_4_C/Ti	Tensile 945 MPa
Li [29]	5 wt.% B4C/Ti	Tensile 1126.1 MPa
This work	30 vol.% core-shell B_4_C/Ti	Compressive 1529.1 MPa

**Table 2:** Functional properties of B_4_C/Ti composites.

Properties	B_4_C/Ti	Ti
Density	4.32 g/cm^3^	4.54 g/cm^3^
Hardness	697.9 HV	210 HV
Thermal conductivity	13.0 W/m·K	14.6 W/m·K
Electric conductivity	0.64 Ω·mm^2^/m	2.34 Ω·mm^2^/m

## Data Availability

The data presented in this study are available on request from the corresponding author.
